# Evaluation of Dietary Management Using Artificial Intelligence and Human Interventions: Nonrandomized Controlled Trial

**DOI:** 10.2196/30630

**Published:** 2022-06-08

**Authors:** Fusae Okaniwa, Hiroshi Yoshida

**Affiliations:** 1 Department of Theoretical Social Security Research National Institute of Population and Social Security Research Tokyo Japan; 2 Graduate School of Economics and Management Tohoku University Miyagi Japan

**Keywords:** health promotion, dietary management, intervention, artificial intelligence, body fat percentage, body mass index, behavioral economics, nonprofessional, Japan

## Abstract

**Background:**

There has been an increase in personal health records with the increased use of wearable devices and smartphone apps to improve health. Traditional health promotion programs by human professionals have limitations in terms of cost and reach. Due to labor shortages and to save costs, there has been a growing emphasis in the medical field on building health guidance systems using artificial intelligence (AI). AI will replace advanced human tasks to some extent in the future. However, it is difficult to sustain behavioral change through technology alone at present.

**Objective:**

This study investigates whether AI alone can effectively encourage healthy behaviors or whether human interventions are needed to achieve and sustain health-related behavioral change. We examined the effectiveness of AI and human interventions to encourage dietary management behaviors. In addition, we elucidated the conditions for maximizing the effect of AI on health improvement. We hypothesized that the combination of AI and human interventions will maximize their effectiveness.

**Methods:**

We conducted a 3-month experiment by recruiting participants who were users of a smartphone diet management app. We recruited 102 participants and divided them into 3 groups. Treatment group I received text messages using the standard features of the app (AI-based text message intervention). Treatment group II received video messages from a companion, in addition to the text messages (combined text message and human video message intervention by AI). The control group used the app to keep a dietary record, but no feedback was provided (no intervention). We examine the participants’ continuity and the effects on physical indicators.

**Results:**

Combined AI and video messaging (treatment group II) led to a lower dropout rate from the program compared to the control group, and the Cox proportional-hazards model estimate showed a hazard ratio (HR) of 0.078, which was statistically significant at the 5% level. Further, human intervention with AI and video messaging significantly reduced the body fat percentage (BFP) of participants after 3 months compared to the control group, and the rate of reduction was greater in the group with more individualized intervention. The AI-based text messages affected the BMI but had no significant effect on the BFP.

**Conclusions:**

This experiment shows that it is challenging to sustain participants' healthy behavior with AI intervention alone. The results also suggest that even if the health information conveyed is the same, the information conveyed by humans and AI is more effective in improving health than the information sent by AI alone. The support received from the companion in the form of video messages may have promoted voluntary health behaviors. It is noteworthy that companions were competent, even though they were nonexperts. This means that person-to-person communication is crucial for health interventions.

## Introduction

Recent years have seen health promotion with the help of technological advancement, such as the use of wearable devices and smartphone apps that record an individual’s health information. However, people struggle to adopt and maintain healthy behaviors [1]. Since 2016, the Japanese government has been providing a large-scale subsidy for the development of artificial intelligence (AI) in the medical field, with emphasis on the use of AI-based personal health records and the creation of health guidance systems. However, as medical and health care AI is still a nascent field, these are yet to be applied in practice and concrete examples explaining their effective use have not been made public yet [2,3].

In this study, we examine the effect of AI intervention on dietary management and elucidate the conditions for maximizing its effectiveness. Focusing on the continuity of health promotion activities and changes in physical indicators, survival analysis, and ordinary least squares (OLS) regression are conducted. We find that for advanced technology to be fully effective, it is necessary to add human intervention and customized care to AI-based interventions. If the incorporation of human interventions significantly improves physical indicators, it suggests that effective AI intervention necessarily requires unique mechanisms with added customized care. Since the COVID-19 pandemic has restricted people to their homes, the value of remote services has increased [4-6]. Online coaching, which links AI and human intervention, encourages healthy behaviors. Similarly, AI intervention could be the initiation of a new business model in this field. It is also an excellent opportunity for people with rare diseases to connect with each other globally. In this study, we focus on the next stage of the challenge in the context of enhancing the outcomes derived from the use of developed technologies. Identifying services that could be more effective with AI intervention will help to drive further technological development and stimulate new demand [7].

Our contributions to this area of study are as follows. First, this study examines the potential effectiveness of AI intervention on health promotion. Previous studies on information and communications technology (ICT) and health interventions have mainly discussed the intervention effects based on text messages [8-10] and health disparities associated with information disparities [11-14]. The main focus of our study is to develop AI-based health intervention systems. The reason for focusing on ICT and health interventions is to examine whether AI alone is sufficient to replace human intervention in this field, and to analyze the elements that can be added to maximize its effectiveness. Specifically, we examine the effectiveness of adding human interventions. Second, we examine the effectiveness of interventions through video messages. Text messages sent through short messaging service (SMS) are useful for health behaviors in the short term [8,10] and result in an increased retention of health behaviors across age and nationality [9]. However, there are not many studies on video message interventions. It should be noted that individuals with lower levels of education might not read the entire text messages [15,16], and those who are less enthusiastic about behavioral change may be more interested in video messages [17]. In previous studies, at the time of the experiments, some participants may have felt uneasy about receiving a video in place of an SMS message or a voice call due to the high cost of roaming data, which can lead to high participant attrition rates that may result in research bias [18,19]. Currently, the introduction of fifth-generation mobile communication system services in developed countries has made video viewing faster and cheaper, which may contribute toward the removal of the bias. In this sense, the effectiveness of video message interventions will increase further in the future. Third, we find that it is relevant to identify the effectiveness of the delivery of video messages by a nonprofessional through customized professional interventions. However, challenges may arise in terms of cost and reach. Exploring the possibility of nonprofessional interventions is a critical perspective when considering measures to combat labor shortages occurring due to a declining population [20]. In epidemiology and clinical fields, these issues have not been extensively studied. Moreover, these issues have not been analyzed from an economic perspective. Computerized messages are costly during development; however, once developed, they are significantly less expensive than human intervention. Moreover, the product has an extensive reach due to its availability through the internet [10,21]. Additionally, health promotion interventions are effective when their content is perceived to be personally relevant [22]. Therefore, in this study, we examine the effectiveness of nonprofessional customized messages with low-cost interventions.

In summary, the purpose of our study is to examine the effectiveness of AI and human interventions to encourage dietary management behaviors. Our study investigates whether AI alone can effectively encourage healthy behaviors or whether human interventions are needed to achieve and sustain health-related behavioral change. We elucidate the conditions for maximizing the effect of AI on health improvement. We hypothesize that the combination of AI and human interventions will maximize their effectiveness.

## Methods

### Standard Specifications of the App

In this study, we used a diet management app for smartphones developed by asken Inc. The app functions in the following way: First, users take pictures of their meal on a smartphone. The app analyzes these pictures, registers the food automatically, and calculates the amount of nutrients and calories in the meal. Additionally, the dietary evaluation score considers ingested nutrients. The app explains its final analysis to its users with the help of graphs and advises them accordingly with a balanced diet and weight loss goals. More than 200,000 pieces of advice are developed from a proprietary algorithm based on a nutritionist’s recommendations and are selected and delivered through unidirectional text messages from the app to the user. The app collects data as it is being used via (1) menus and intake of calories and nutrients, (2) dietary evaluation scores by a nutritionist in the system, (3) weight and body fat percentage (BFP) voluntarily registered by survey participants, and (4) profiles (age, gender, and height) of participants who undertake the survey.

### Interventions

**Figure 1:**
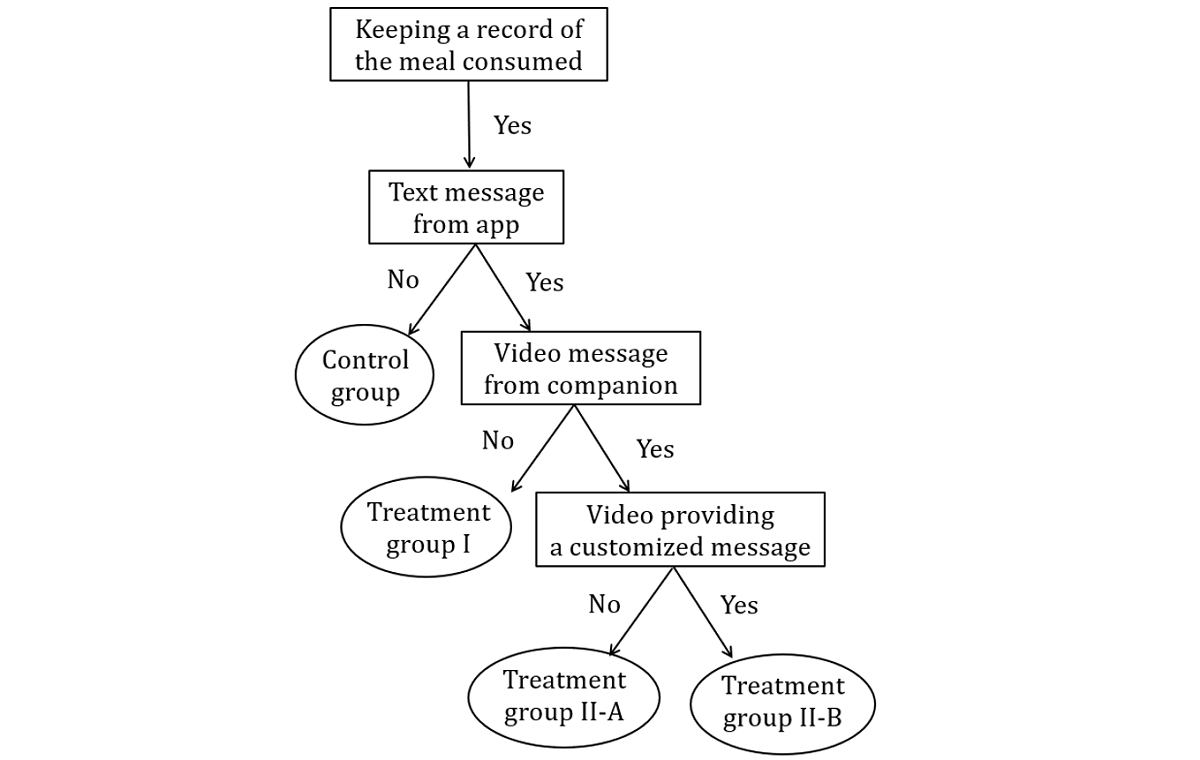
Type of intervention.

When we recruited the users of the app as the participants of our study with the cooperation of asken Inc, 102 app users voluntarily participated in our experiment. They committed to record their meals on weekdays. The reason for this was to reduce the burden on participants. However, to not interfere with the behavior of motivated participants who wanted to record their holidays, we made it possible to voluntarily record their holidays. However, these holiday data were not used in this study. We promised the participants at the beginning of the experiment that we would use the weekday data. We also considered that the meals on holidays might be different from those on weekdays, but this would be reflected in the data after Monday. We conducted the experiment over a period of 3 months from February to April 2020. As shown in Figure 1, we divided the 102 app users in our study into 3 groups using the following interventions:

- First, in treatment group I, the standard features of the app were used. In other words, the participants recorded the dietary details of what they consumed in the app. Depending on their diet, the AI provided advice on a balanced diet from a list of 200,000 suggestions created by a confidential algorithm. For example, if a participant eats only 1 bowl of soup and records the contents in the app, it provides the following text advice: “Your meal balance score is 50. Your calorie intake is insufficient. In terms of nutritional balance, you are particularly deficient in protein and carbohydrates. For your next meal, please increase your overall food intake, focusing on meat and fish. Be sure to include 1 serving of bread or rice.” The app also provides graphs of nutrient intake and target values. These are provided via text messages.
- Second, treatment group II was provided with the same intervention as treatment group I, with the addition of a video of a human reading a text message by the app. Participants recorded their dietary details, reviewed the text advice provided by the app, and then took a screenshot of the text advice on their smartphone. They were then required to send the screenshot to their companion using the LINE app. The specifications of the app did not allow the companion advance knowledge of the text advice that would be provided to the participant. The companion was required to confirm the content of the advice on the LINE app, create a video message with an oral and visual description of the advice, and send it back to the participant. This series of events was the intervention for treatment group II. The companion is a nonprofessional, and the message is not beyond the advice of a nutritionist. They act as a human intervention to the AI text message.
- Third, we used a control group that used the app to record their meals, but no advice was delivered to them during our experiment. Therefore, participants in the control group took care of their diet and health based on their records.

Given the results of the first 2 months of the experiment, we introduced another intervention during the third month. Treatment group II was further divided into 2 subgroups: treatment group II-A received the same intervention as before, and treatment group II-B received customized care. In the former case, the companion read out a part of the text message through AI intervention. However, in the case of the latter, the companion graphed the dietary evaluation scores and provided encouraging messages based on each person's efforts. Moreover, when participants sent us online messages expressing their impressions, the companion responded to them with a video message and delivered flexible and personalized messages.

Participants were allowed to choose which group they wanted to participate in. Treatment group I used the standard specifications of the app, meaning that there was no new burden for participants who were already using the app. Participants in treatment group II, however, were required to perform additional operations related to taking and sending screenshots, which was a new burden. We determined that it was necessary to let the participants decide for themselves whether to accept this burden.

### Outcome

To understand the effects of the intervention, we analyzed 2 outcomes: (1) continuity of the program and (2) physical indicators. We used the BFP and BMI as physical indicators. In the questionnaire survey regarding the purpose of using the app, most participants responded with “to develop good eating habits” and “to lose weight.” Therefore, as outcomes, the effect of “good eating habits” was confirmed by continuity and the effect of weight loss was confirmed by the BFP and BMI [23].

We used the number of meals recorded by the participants as a measure of continuity. The experiment’s total duration was 13 weeks. We recorded their meals for more than 5 days consecutively, excluding holidays. If the participants did not record their meals for even 5 days, they were regarded as dropouts.

The BMI is a measure of nutritional status in adults. Although it is a simple indicator that can be calculated from the height and weight of an individual alone, it is not a complete physical status indicator, because it does not predict the composition of a body accurately [24,25]. However, the BFP is a more reliable health indicator than the BMI because it calculates fat as a percentage of body weight and represents its composition. The American College of Physicians has stated that the BFP is more important than the BMI in assessing a patient’s health and mortality risk [26]. Therefore, we have used the BFP as an essential health indicator in this study. Additionally, the BMI was used as a supplement to measure short-term effects. We used the average of the first week of our study as a reference point and focused on the rate of change after 3 months. As we changed the methods of treatment group II in the last month of our study, we also examined the rate of change in the average value, comparing the last week of March to the last week of April. The descriptive statistics for each variable are shown in Table S1 in Multimedia Appendix 1.

### Analytical Approach

We analyzed the effects of the intervention on continuity and individuals' health indicators. First, for continuity of health behaviors, we executed a survival analysis. We regarded dropouts in our experiment as deaths, as in the case of a general survival analysis. The number of dropouts is shown in Table 1. First, we used a nonparametric model (Kaplan-Meier analysis) that did not assume a specific distribution for survival time and did not examine the effects of the covariates. We also analyzed the data with a semiparametric model (Cox proportional-hazards model [27]) that did not assume a specific distribution on survival time but estimated the parameters of the covariates and examined an effect on survival time. We created dummy variables for each group and examined the effects of each intervention. The Cox proportional-hazards model is expressed as

h(t|T_ij_, X_i_) = H_0_(t) exp(β_ij_T_ij_ + γ_ik_X_i_),

where h_0_(t) is the baseline hazard; “i” denotes the participant number; “j” denotes the treatment group number; β and γ are hazard ratios (HRs); T is the treatment group dummy; and X is the set of control variables (age, gender, height).

Second, we conducted OLS regressions for health indicators:

R_ik_ = α_i_ + δ_ij_T_ij_ + θ_i_X_i_ + ε_i_

where R_k_ is the rate of change in the BFP (k=1) or the BMI (k=2) in experimental periods; δ and θ are coefficients vectors; and ε is an error term. We focused on the changes after 3 months. Moreover, we examined the effects of changing the intervention method for treatment group II during the last month.

**Table 1:** Number of dropouts and test for equality of survivor functions. Results of the estimation using a nonparametric model (Kaplan-Meier model) were as follows: Pr>χ2=0.012 (log-rank test) and 0.011 (Wilcoxon test), where Pr refers to probability.

		Control group (N=34)	Treatment group I (N=34)	Treatment group II (N=34)
**Week^a^, n (%)**				
	1	1 (3)	N/A^b^	N/A
	2	1 (3)	1 (3)	N/A
	3	N/A	1 (3)	N/A
	4	1 (3)	N/A	N/A
	5	N/A	N/A	N/A
	6	N/A	N/A	N/A
	7	1 (3)	2 (6)	N/A
	8	1 (3)	N/A	N/A
	9	N/A	2 (6)	N/A
	10	2 (6)	2 (6)	N/A
	11	2 (6)	N/A	N/A
	12	N/A	2 (6)	1 (3)
	13	N/A	N/A	N/A
Total dropouts, n (%)		9 (27)	10 (29)	1 (3)
Dropouts expected, n (%)		6.32 (19)	6.37 (19)	7.32 (22)
Sum of ranks		258	337	–595

^a^The experiment’s total duration was 13 weeks (from February to April), and participants were considered to have dropped out of the program if they did not record their meals for more than 5 days consecutively, excluding holidays.

^b^N/A: not applicable.

### Ethical Approval

The research ethics committee of the Graduate school of Tohoku University approved this study (approval date: January 31, 2020). We experimented with a noncontact and noninvasive approach. Recruitment was conducted by asken Inc on our behalf, and we did not have any contact with the participants. This study sought to collect and analyze information registered on the app with the permission of the participants. Necessary and sufficient information was provided to the participants, and asken Inc obtained consent from them through the app before providing the data to us. The advice communicated by the companion did not exceed the scope of the textual advice independently generated by the app designed based on a nutritionist’s advice. There were no new risks inherent in this experiment, and the safety of the experiment was ensured.

## Results

### Effects on Continuity

**Figure 2:**
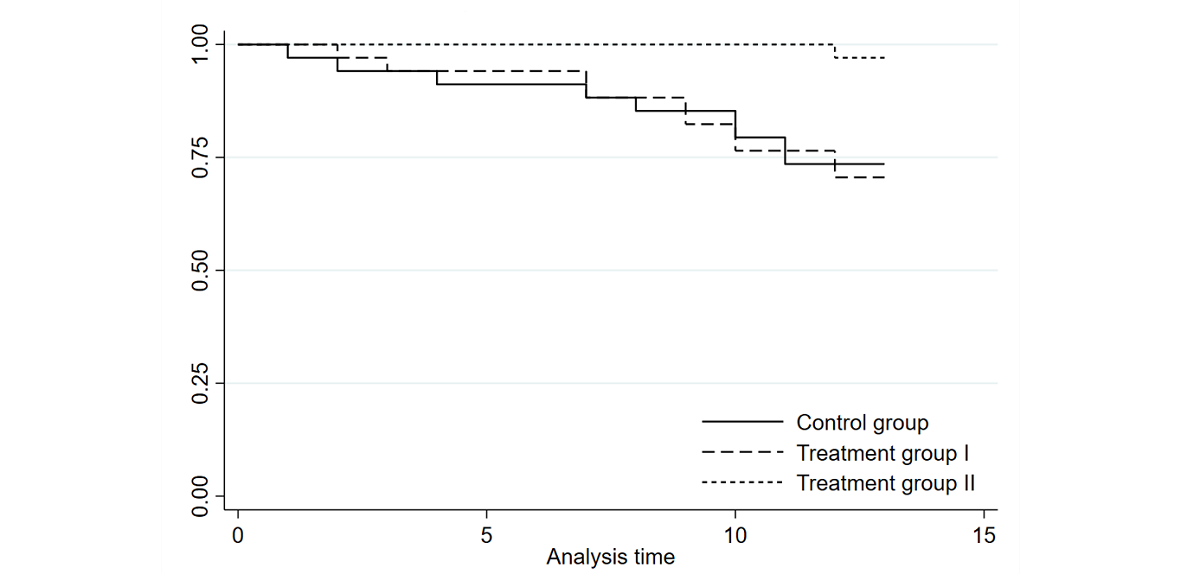
Kaplan-Meier survival estimates. Treatment group I received a text message intervention by AI, and treatment group II received a customized video message intervention along with the text message intervention. AI: artificial intelligence.

Figure 2 shows the survival curve based on the Kaplan-Meier model. The vertical axis is the survival rate (here representing continuity), and the horizontal axis is the duration of the experiment (weeks). Treatment group II had the highest survival rate, followed by the control group and treatment group I. The survival curves of the 3 groups were examined for statistical significance. Results of the generalized Wilcoxon test and the log-rank test showed a significant difference between the 3 groups’ survival curves at the 5% level of significance (Table 1).

Additionally, we estimated a Cox proportional-hazards model to account for the effect of the covariates (Table 2). The number of observations was 102, the number of failures was 20, and the times at risk were 1223. The HR for treatment group II was statistically significant at 0.078, that is, the dropout rate was 0.078 times higher (92% reduction) compared to the control group. This means that treatment group II had 92% fewer dropouts compared to the control group. However, for treatment group I, the HR was <1 but not statistically significant. Thus, receiving only text messages (treatment group I) does not represent a significant difference in persistence compared to receiving no intervention (control group).

**Table 2:** Results of HRs^a^ from the Cox proportional-hazards model (Pr>χ2=.009).

	HR	95% CI	*P* value
Treatment group I^b^	0.975	0.391	.96
Treatment group II^c^	0.078	0.009	.02
Age (years)	0.970	0.930	.17
Male	2.423	0.643	.19
Height	0.941	0.863	.17

^a^HR: hazard ratio.

^b^Treatment group I received a text message intervention by artificial intelligence.

^c^Treatment group II received a video message intervention along with the text message intervention.

### Effects on Physical Indicators

After 3 months (Table 3), the BFP of treatment group II significantly decreased, but it was not statistically significant compared to treatment group I. However, the BMI was statistically significant for treatment group I but insignificant for treatment group II after 3 months. As there was no significant difference in human intervention during the first 2 months, we introduced another intervention during the third month. Treatment group II was further divided into 2 subgroups: treatment group II-A received the same intervention as before, and treatment group II-B received customized care. Observing the effects of the change in the intervention method in the last 1 month on treatment group II showed that both treatment groups II-A and II-B were statistically negatively significant for the BFP. The reduction was higher for treatment group II-B, which received a more customized intervention. However, the reduction for treatment group I was not statistically significant with AI intervention. For the BMI in the last 1 month, neither treatment group II-A nor treatment group II-B was statistically significant but treatment group I was statistically negatively significant. The number of observations during this period was as follows: BFP (3 months), 35; BMI (3 months), 64; BFP (last 1 month), 34; and BMI (last 1 month), 60. In addition, the adjusted *R^2^* values were 0.009, 0.072, 0.170, and 0.057, respectively.

Indicators of the effect size can be derived by several methods of analysis, such as the *t* test, ANOVA, and multiple regression analysis. In this study, multiple regression analysis was used to include more information in the estimation, and we used adjusted *R^2^* as an indicator of the effect size. Only the effect size of the last month’s BFP was medium, but all other effect sizes were small [28]. In addition, we controlled for age and gender as personal characteristics that could affect the outcome. The physical activity level could not be included. The data we were able to collect from the app were rough, and therefore, there was little difference between participants.

**Table 3:** Regression results of physical indicators (OLS^a^ estimation).

	After 3 months							Last 1 month				
	BFP^b^			BMI			BFP				BMI	
	Coefficient (SE)	*P* value	Coefficient (SE)		*P* value	Coefficient (SE)			*P* value	Coefficient (SE)		*P* value
Treatment group I^c^	–0.029 (0.056)	.61	–0.026 (0.010)		.44	–0.013 (0.045)			.77	–0.009 (0.004)		.04
Treatment group II^d^	–0.102 (0.050)	.05	–0.008 (0.010)		.01	N/A			N/A	N/A		N/A
Treatment group II-A^e^	N/A^f^	N/A	N/A		N/A	–0.082 (0.044)			.07	–0.003 (0.005)		.50
Treatment group II-B^g^	N/A	N/A	N/A		N/A	–0.218 (0.074)			.01	0.007 (0.009)		.43
Male	0.031 (0.048)	.52	–0.011 (0.009)		.22	0.028 (0.041)			.49	–0.007 (0.004)		.10
Age (years)	–0.001 (0.002)	.59	0.000 (0.000)		.31	–0.001 (0.002)			.78	0.000 (0.000)		.75
Constant	0.019 (0.110)	.86	0.013 (0.017)		.46	0.027 (0.090)			.77	0.006 (0.008)		.51

^a^OLS: ordinary least squares.

^b^BFP: body fat percentage.

^c^Treatment group I received a text message intervention by artificial intelligence.

^d^Treatment group II received a video message intervention along with the text message intervention. During the last month, treatment group II was divided into 2 subgroups.

^e^Treatment group II-A received a video intervention that only read out text messages as before.

^f^N/A: not applicable.

^g^Treatment group II-B received a more customized intervention.

## Discussion

### Principal Findings

A society in which individuals use a variety of new devices to engage in health promotion activities in their daily lives was envisioned in this study. To effectively implement advanced technologies in health promotion, it is important to know what interventions might encourage people to engage in autonomous health activities. We examined the effectiveness of interventions by AI and humans to encourage healthy behaviors. Our study compared continuity of the program and the effects on health indicators using an AI-based intervention, with text messages and customized (nonexpert) video messages added to the intervention for the users of a dietary management app. We found that combined AI and video messaging (treatment group II) led to a lower dropout rate from the program compared to the control group, and the Cox proportional-hazards model estimate showed an HR of 0.078, which was statistically significant at the 5% level. Further, human intervention with AI and video messaging significantly reduced the BFP of participants after 3 months compared to the control group, and the rate of reduction was greater in the group with more individualized intervention. The AI-based text messages affected the BMI but had no significant effect on the BFP.

### Considerations and Future Directions

First, this study shows that it is challenging to sustain healthy behaviors with AI intervention alone. We also found that health improvement is the highest when the intervention is delivered with human video messages and AI text messages. Traditional economic theory assumes that people behave rationally after receiving accurate information. Behavioral economics, however, calls this rational world bias and considers that, in reality, it is challenging to take a rational action [29-31]. The results of this study suggest the existence of a rational world bias in ongoing healthy behavior and long-term health improvement; providing information through AI alone may only have short-term effects.

Second, adding human intervention to AI intervention by delivering videos of an individual reading AI-based text messages (treatment group II-A) had a significantly positive impact on persistence and health promotion effects. This means that even if the information conveyed is the same, differences in the delivery method produces differences in effectiveness. Here, human-communicated information was more effective in promoting healthy behavior.

Third, the effect was higher when the companion increased the level of customization, such as by mentioning the participants' names in the videos while cheering them on and plotting the trends in a graph in their respective dietary evaluation scores (treatment group II-B). This can be considered a type of coaching effect. Highly individualized interventions are effective [32], and direct coaching is also effective [33]. Further, this study confirmed the effectiveness of remote coaching.

Fourth, it is also important to note that the companions were capable, even though they were not professionals in the field. This means that the process of human communication is as important as the content of the information. Due to the effectiveness of nonprofessional interventions, there is potential to address labor shortages due to a decline in the future population and human costs.

Fifth, behavioral economics believes that humans are limitedly rational beings and tend to have base rate neglect [34]. For example, even if obesity is known to increase the risk of acquiring diseases in the future, it is difficult to continue health behaviors. This may be especially true for people with high time preference, that is, individuals who prioritize present utility over future utility. In this experiment, nonexperts supporting users every day increased the continuity effect. For those with high time preference rates, the daily support received from their companions may have been a reward and an incentive to continue their long-term health behaviors. It may be useful to consider effective interventions for people with high time preference rates who have difficulty sustaining healthy behaviors.

Finally, we also infer that each intervention has different effects on the BFP and BMI. The BMI is calculated based on the height and weight of individuals only, and it cannot distinguish between fat and muscle. Therefore, it is an indicator that quantifies the apparent body size. The height of an adult does not change immediately, but the weight fluctuates with the content and frequency of meals per day, which can result in an increase or decrease in the BMI. However, the BFP is a measure of body composition; it cannot be reduced immediately and requires continuous effort. This difference is evident from the fact that the correlation between BMI and BFP is not always strong [35]. The AI text messages had a significant and negative effect on the BMI but no statistically significant effect on the BFP. This suggests that text messages may have effectively promoted weight loss in the short term, but thi may not hold true in the long term. On the contrary, customized video messages did not affect the BMI and had a significantly negative effect on the BFP, which implies that the video messages were effective in promoting ongoing health behaviors.

### Limitations

There are several limitations to the interpretation of our results. First, there exists a sampling bias. We recruited app users as participants and also asked them to choose the intervention group they wanted to belong to. These aspects may have affected our results. Second, participants may have been highly conscious about their health; hence, the results of our study may be valid only for those who are already health conscious. The effect on people with low health consciousness requires further study. Owing to limitations in the data we collected, we were not able to include in our estimates many confounding variables that could affect outcomes, and the effect size was also small. Approximately 100 users participated in our study, which is not a sufficient size; hence, it may be difficult to generalize the results obtained.

Second, since weight and BFP data were obtained voluntarily, only highly voluntary and health-conscious individuals may have recorded the data. On the last day of the experiment, 60 (58.8%) of the 102 participants continued recording their weights and 34 (33.3%) continued recording their BFP. However, dietary records continued at 82 (80.4%) of the 102 participants in the last month. Therefore, bias may have occurred due to voluntary collection of data. AI and human interventions involved a procedure in which participants were required to send a screenshot of a text message to the companion. The procedure was carried out because our experiment was conducted without changing the specifications of the app. This may have increased the spontaneity of the participants.

Third, we should note the validity of the outcome. The app used in the experiment and the advice provided were designed to shape a balanced diet. Therefore, it is plausible to use an indicator that is related to diet quality. The app delivers the dietary evaluation scores calculated by the proprietary algorithm of asken Inc via text messages. However, we were unable to confirm the objective validity of this score. The results of our survey showed that the participants were interested in healthy living in a broader sense and not by merely being on a balanced diet. Therefore, we used objective health indicators as outcomes. The collection of adequate objective data on dietary quality is a challenge for future research.

Finally, the analysis did not include the costs of human interventions. In our study, the combination of AI and human interventions had the highest effect. However, human intervention is costly. No matter how significant the effect is, the behavioral change may not necessarily sustain if users and companions pay high costs (eg, in terms of money, time, human resources, and efforts) to take advantage of it. Therefore, it is necessary to examine human intervention in conjunction with the costs.

### Conclusion

This experiment shows that it is challenging to sustain participants' healthy behavior with AI intervention alone. The results also suggest that even if the health information conveyed is the same, the information conveyed by humans is more effective in improving health than the information sent by AI. The support received from the companion in the form of video messages may have promoted voluntary health behaviors. It is noteworthy that the companions were competent, even though they were nonexperts. This means that the process of person-to-person communication is crucial for health interventions. The results of this experiment show both short- and long-term effects, which may help us consider effective intervention strategies that respond to these differences with respect to time preference.

## Appendix

Multimedia Appendix 1 Table S1. Description of variables.

## Abbreviations

AI: artificial intelligence
BFP: body fat percentage
HR: hazard ratio
ICT: information and communications technology
OLS: ordinary least squares
SMS: short messaging service

## Data Availability

The data sets generated and analyzed in this study are not publicly available due to confidentiality and ethics.
