# Supplementary material for: Evaluation of Dietary Management Using Artificial Intelligence and Human Interventions: Nonrandomized Controlled Trial
Source: JMIR Form Res. 2022 Jun 8;6(6):e30630. doi: 10.2196/30630 (PMC9218879; doi:10.2196/30630)
Supplement: Multimedia Appendix 1 [file formative_v6i6e30630_app1.docx]

Table S1. Description of variables

| **Variable** | Control group | | | | | Treatment (I) | | | | | Treatment (II) | | | | |
| --- | --- | --- | --- | --- | --- | --- | --- | --- | --- | --- | --- | --- | --- | --- | --- |
|  | Obs. | Mean | Std. Dev. | Min | Max | Obs. | Mean | Std. Dev. | Min | Max | Obs. | Mean | Std. Dev. | Min | Max |
| Male | 34 | 0.32 | 0.47 | 0.00 | 1.00 | 34 | 0.29 | 0.46 | 0.00 | 1.00 | 34 | 0.50 | 0.51 | 0.00 | 1.00 |
| Age | 34 | 39.38 | 9.46 | 17.00 | 59.00 | 34 | 36.44 | 10.55 | 16.00 | 59.00 | 34 | 36.09 | 11.72 | 18.00 | 60.00 |
| Record of 1st month | 34 | 26.94 | 5.10 | 7.00 | 29.00 | 34 | 27.91 | 4.24 | 7.00 | 29.00 | 34 | 28.06 | 1.95 | 21.00 | 29.00 |
| Record of 2nd month | 34 | 24.32 | 9.30 | 0.00 | 29.00 | 34 | 25.38 | 7.27 | 2.00 | 29.00 | 34 | 26.18 | 4.16 | 16.00 | 29.00 |
| Record of 3rd month | 34 | 23.06 | 11.58 | 0.00 | 30.00 | 34 | 21.71 | 11.37 | 0.00 | 30.00 | 34 | 25.85 | 4.38 | 14.00 | 30.00 |
| Height | 34 | 163.14 | 7.32 | 153.00 | 177.50 | 34 | 163.16 | 7.37 | 150.00 | 177.20 | 34 | 165.36 | 8.34 | 151.00 | 186.00 |
| Weight in 1st week | 34 | 61.68 | 11.06 | 37.55 | 87.67 | 33 | 66.94 | 17.01 | 45.57 | 106.31 | 32 | 70.09 | 11.17 | 49.74 | 93.56 |
| Body fat in 1st week | 18 | 28.73 | 4.89 | 19.33 | 38.10 | 18 | 29.96 | 8.68 | 15.12 | 48.81 | 21 | 28.31 | 7.76 | 11.80 | 46.57 |
| Weight in 13th week | 24 | 60.26 | 8.90 | 46.13 | 76.35 | 21 | 63.82 | 16.02 | 45.00 | 95.91 | 19 | 67.10 | 11.20 | 47.21 | 85.48 |
| Body fat in 13th week | 17 | 27.62 | 6.04 | 18.09 | 37.53 | 7 | 30.26 | 9.34 | 17.30 | 47.18 | 13 | 23.45 | 7.74 | 11.91 | 43.77 |

Source: Authors' survey (based on the intervention)
